# Phosphorylation of S122 in ERα is important for the skeletal response to estrogen treatment in male mice

**DOI:** 10.1038/s41598-022-26939-9

**Published:** 2022-12-27

**Authors:** Karin Horkeby, Helen H. Farman, Sofia Movérare-Skrtic, Vikte Lionikaite, Jianyao Wu, Petra Henning, Sara Windahl, Klara Sjögren, Claes Ohlsson, Marie K. Lagerquist

**Affiliations:** 1 Sahlgrenska Osteoporosis Centre, Centre for Bone and Arthritis Research at Institute of Medicine, Sahlgrenska Academy at University of Gothenburg, Klinfarmlab, Vita Stråket 11, 413 45 Göteborg, Sweden; 2 Division of Pathology, Department of Laboratory Medicine, Karolinska Institute, Huddinge, Sweden; 3 Department of Drug Treatment, Sahlgrenska University Hospital, Region Västra Götaland, Gothenburg, Sweden

**Keywords:** Endocrinology, Osteoporosis

## Abstract

Estrogen receptor alpha (ERα) signaling has beneficial skeletal effects in males. ERα signaling also affects other tissues, and to find bone-specific treatments, more knowledge regarding tissue-specific ERα signaling is needed. ERα is subjected to posttranslational modifications, including phosphorylation, which can influence ERα function in a tissue-specific manner. To determine the importance of phosphorylation site S122 (corresponding to human ERα site S118) for the skeleton and other tissues, male mice with a S122A mutation were used. Total areal bone mineral density was similar between gonadal intact S122A and WT littermates followed up to 12 months of age, and weights of estrogen-responsive organs normalized for body weight were unchanged between S122A and WT males at both 3 and 12 months of age. Interestingly, 12-month-old S122A males had decreased body weight compared to WT. To investigate if site S122 affects the estrogen response in bone and other tissues, 12-week-old S122A and WT males were orchidectomized (orx) and treated with estradiol (E2) or placebo pellets for four weeks. E2 increased cortical thickness in tibia in both orx WT (+ 60%, p < 0.001) and S122A (+ 45%, p < 0.001) males. However, the E2 effect on cortical thickness was significantly decreased in orx S122A compared to WT mice (− 24%, p < 0.05). In contrast, E2 affected trabecular bone and organ weights similarly in orx S122A and WT males. Thus, ERα phosphorylation site S122 is required for a normal E2 response specifically in cortical bone in male mice, a finding that may have implications for development of future treatments against male osteoporosis.

## Introduction

Estrogen affects several organs and physiological functions in both women and men including reproductive organs, the immune system, weight regulation and energy metabolism^1–3^. The skeleton is also an estrogen-responsive organ, and the importance of estrogen for the skeleton in women is well established^4^. In recent decades, the importance of estrogen for male skeletal health has been increasingly recognized. Human and rodent studies have shown that estrogen is involved in the regulation of bone development and maintenance in males^5–10^. Furthermore, serum estradiol (E2) levels have been shown to be strongly associated with both bone mineral density (BMD) and fracture risk in men^11–14^. Estrogen deficiency, which leads to decreased bone mass after menopause in women, is even proposed to be the main cause of male age-related bone loss^9,15,16^. In addition, we recently demonstrated causal effects of serum E2 levels on both BMD and fracture risk in men using mendelian randomization^17,18^. Estrogen has beneficial effects on the skeleton in men but is not used clinically to prevent bone loss because of its feminizing effects. Development of bone-specific estrogen treatments with minimal adverse effects in other tissues may aid the prevention and treatment of osteoporosis in an aging male population. To achieve this, it is important to further characterize estrogen signaling in bone and other tissues in males. Estrogen receptor α (ERα) mediates estrogenic effects in the male skeleton as demonstrated in humans and in animal studies^19–22^. The function of ERα is activated by ligand binding and regulated by post-translational modifications (PTMs), including phosphorylation^23^. ERα can be phosphorylated on multiple amino acids throughout the whole protein and within all major structural domains^24^. A number of these phosphorylation sites have been characterized in vitro and shown to regulate various functional activities such as hormone sensitivity, nuclear localization, DNA binding, protein/chromatin interactions, protein stability, and transcriptional activity^25–31^. Serine 118 (S118, corresponding to S122 in the murine ERα), a well-characterized phosphorylation site in the human ERα, is a highly conserved residue located in the N-terminal A/B domain where activation function 1 (AF-1) is located. The importance of S118 phosphorylation has been studied in vitro in different laboratories by using a mutant ERα, where the serine is mutated to alanine (S118A-ERα), which renders the ERα unable to be phosphorylated at this site^25,32^. Interestingly, the inability of the human ERα to be phosphorylated at site 118 leads to decreased estradiol‐induced gene transcription in some^26^ but not all^33^ cell‐types, suggesting that the importance of this phosphorylation site may differ in a tissue‐dependent manner. We recently investigated the role of ERα phosphorylation site S122 in vivo in female mice, using a novel S122A genetic mouse model that lacks the ability to phosphorylate site 122 of ERα, and reported that the S122A mutation selectively affects the metabolism in females, leading to increased fat mass and insulin levels^34^. Thus, the phosphorylation site S122 in ERα has a tissue-dependent role in vivo in females. The aim of this study was to investigate if phosphorylation site S122 of ERα has a tissue-specific role also in males by 1) comparing weights of various estrogen-responsive tissues and body composition-related parameters between WT and S122A mice at different timepoints and 2) comparing estrogen treatment responses between WT and S122A mice in the estrogen-responsive tissues and body composition-related parameters.

## Material and methods

### Animals

The generation of S122A mice, with a point mutation in ERα resulting in a single amino acid substitution from serine to alanine at site S122, and littermate wild type (WT) mice has previously been described^34^. The following primers were used for genotyping of the S122A mice: 5′-GGGAAGTCAGAAAGGATTCTTTGGCAG-3′ and 5′-GGTTCCTTGCTAGCACAGGCCAC-3′. The mice were housed in a standard animal facility under controlled temperature (22 °C) and photoperiod (12 h of light and 12 h of darkness) and fed phytoestrogen free pellet diet ad libitum (Rodent diet Harlan 2016). Gonadal intact S122A and WT littermate male mice were terminated at three months of age (young adult). In a second experiment, middle-aged mice were followed between six and twelve months of age and then terminated. For assessment of the E2 response, three-month-old male mice were orchidectomized (orx) and treated subcutaneously with slow-release pellets (60-day-release pellet, Innovative Research of America) with 17β-estradiol (E2, 16.7 ng/mouse day) or corresponding placebo pellet for four weeks. For assessment of the effect of orx, a sham-operated group, treated with placebo pellet, was included. The mice were randomly divided into treatment groups based on the baseline body weight. The WT and S122A mice were mixed in the cages to avoid confounding. Surgery was performed under anesthesia with isoflurane (Baxter Medical AB, Kista, Sweden) and Rimadyl (Orion Pharma AB, Animal Health, Sollentuna, Sweden) was given postoperatively as an analgesic. At termination, the mice were anesthetized with Ketanest/Dexdomitor (Pfizer/Orion Pharma), bled, and euthanized by cervical dislocation. Liver, thymus, testes, and fat depot were collected and weighed. Tibiae were dissected, fixated in 4% paraformaldehyde for two days and stored in 70% ethanol for further analysis. Femora were dissected and flushed with PBS and the cortical bone was snap-frozen and stored in − 80° for further analysis. All experimental procedures involving animals were approved by the Ethics Committee in Gothenburg (ethic numbers 46-2014, 147-2015) and performed according to relevant guidelines. The studies are reported according to ARRIVE guidelines.

### Serum measurements

Serum levels of dihydrotestosterone (DHT), testosterone, and estradiol were measured in three-month-old S122A and WT mice with a single run by gas chromatography-tandem mass spectrometry (GC–MS/MS), as described previously^35^. The sensitivity (lower limit of detection) values for the used high sensitivity gas chromatography-tandem mass spectrometry (GC–MS/MS) were as follows: DHT; 1.6 pg/ml, testosterone; 4.0 pg/ml, estradiol; 0.3 pg/ml. Serum levels of leptin were measured in twelve-month-old S122A and WT males using a Mouse Leptin ELISA kit (cat# 90 030, Chrystal Chem, Zaandam, Netherlands).

#### Assessment of bone parameters

#### Dual-energy X-ray absorptiometry (DXA)

Analyses of total body areal bone mineral density (aBMD), lean mass, and fat percent were performed using the Lunar PIXImus mouse densitometer (Wipro GE Healthcare).

#### Peripheral quantitative computed tomography

Peripheral Quantitative Computed tomography (pQCT) scans were performed with the pQCT XCT RESEARCH M (version 4.5B, Norland, Fort Atkinson, WI) operating at a resolution of 70 μm, as described previously^10,36^. Trabecular BMD was determined ex vivo, with a metaphyseal pQCT scan of the proximal tibia. The scans were positioned in the metaphysis at a distance from the growth plate, corresponding to 2.6% of the total length of the tibia. The trabecular bone region was defined as the inner 45% of the total cross-sectional area. Cortical bone parameters were analyzed in the mid-diaphyseal region of the tibia.

#### High-resolution microcomputed tomography

High-resolution microcomputed tomography (μCT) analysis was performed in the proximal tibia using an 1172 model μCT (Bruker MicroCT, Aartselaar, Belgium) as previously described^37^. The cortical measurements were performed in the mid-diaphyseal region of the tibia starting at a distance of 5.2 mm from the proximal growth plate and extending a further longitudinal distance of 134 μm in the distal direction. The trabecular bone distal to the proximal growth plate was selected for analyses within a conforming volume of interest (cortical bone excluded), commencing at a distance of 650 μm from the growth plate and extending a further longitudinal distance of 134 μm in the distal direction.

#### Mechanical strength

Before mechanical testing, the bones were rinsed in PBS. The three-point bending test (span length 5.5 mm, loading speed 0.155 mm/s) at the mid tibia was made by the Instron universal testing machine (Instron 3366; Instron Corp., Norwood, MA). Based on the recorded load-deformation curves, the biomechanical parameters were acquired from raw files produced by Bluehill 2 software version 2.6 (Instron) with custom-made Excel macros.

### Quantitative real-time PCR analysis

Total mRNA from femur cortical bone was prepared using TriZol (Life Technologies) and RNeasy Mini Kit (Qiagen). The mRNA was reverse transcribed into cDNA (Applied Biosystems) and real-time PCR analyses were performed using the StepOnePlus Real-Time PCR System (Thermo Fischer Scientific). The Assay-on-Demand primer and probe set for Estrogen receptor alpha (*Esr1*: Mm00433147_m) was used. The expression was normalized to the 18S subunit (4310893E). The relative gene expression values were calculated using the ΔΔCt method.

### Statistical analyses

All values are given as mean ± SEM. The statistical differences between WT and S122A in the gonadal intact experiments terminated at three and twelve months of age were calculated using unpaired Student’s *t*-test (Microsoft Excel, version 16.59). For the longitudinal data, a two-way ANOVA (GraphPad Prism, version 9.3.1) was used to determine the presence of statistical differences between WT and S122A mice over time, and posthoc analysis (Šidák´s multiple comparisons test) was used to determine differences at the different timepoints. To analyze differences in E2 treatment or orx responses between WT and S122A mice, the interaction factor from a two-way ANOVA was used, and treatment/orx effects in WT and S122A mice was analyzed by Šidák´s multiple comparisons test. A difference was considered significant when p < 0.05. Power analysis using P*Power 3.1 indicated that a minimum of 8 mice per group provides > 80% power to detect a 1.4 SD difference between groups. Actual sample sizes are indicated in the figure and table legends.

## Results

### Gonadal intact S122A male mice have normal sex steroid levels and a normal skeleton, but decreased body weight

In young adult gonadal intact mice, there was no difference between S122A and WT mice in the mRNA expression level of ERα measured in cortical bone (Table 1). Serum levels of testosterone and DHT, measured by GC–MS/MS, were not altered in S122A males compared to WT littermates, demonstrating a normal sex hormone negative feedback regulation in male mice lacking phosphorylation site S122, and serum levels of E2 were undetectable in both S122A male mice and WT littermates (Table 1). To investigate the role of phosphorylation site S122 in ERα for the physiological regulation of the skeleton in young adult males, we studied gonadal intact S122A male mice at the age of three months. DXA and pQCT measurements showed no differences in total body aBMD, cortical thickness, or trabecular BMD in tibia between S122A and WT mice (Table 1). The organ weights per body weight including liver, thymus, and testes, as well as lean mass measured by DXA, were comparable between S122A and WT male mice (Table 1). Body weight, fat percent as measured by DXA, and dissected gonadal fat weight per body weight were unaltered between young adult S122A and WT males, as reported before^34^, and the total body fat mass, (body weight x fat percent), was also similar between S122A and WT males (Table 1).

**Table 1:** Description of three-month-old male S122A (n = 8) and wild-type (WT, n = 11) littermates.

	WT	S122A
ERα expression in cortical bone (AU)	3.3 ± 0.4	3.3 ± 0.4
Testosterone (pg/ml)	2771 ± 1821	2299 ± 1808
DHT (pg/ml)	61.9 ± 28.6	59.2 ± 36.4
E2 (pg/ml)	nd	nd
Total body aBMD (mg/cm^2^)	50.1 ± 0.6	51.0 ± 0.9
Tibia cortical thickness (μm)	193.1 ± 4.7	206.1 ± 6.4
Tibia trabecular BMD (mg/cm^3^)	249.5 ± 16.3	300.1 ± 25.3
Liver weight/bw (mg/g)	48.7 ± 0.9	46.1 ± 3.1
Thymus weight/bw (mg/g)	1.40 ± 0.04	1.29 ± 0.06
Testis weight/bw (mg/g)	7.6 ± 0.2	7.1 ± 0.2
Lean mass (g)	20.9 ± 0.6	21.9 ± 0.2
Total body fat mass (g)	4.4 ± 0.3	4.0 ±0.5

ERα mRNA expression was measured by real-time PCR. Sex steroid levels were measured with GC–MS/MS. Body composition (total body aBMD, lean mass and total body fat mass) was measured by DXA, and bone parameters (trabecular BMD and cortical thickness) were analysed by pQCT. Values are given as mean ± SEM. Student's t-test, WT vs S122A mice. AU; arbitrary unit, nd; not detected, bw; body weight.

**Figure 1:**
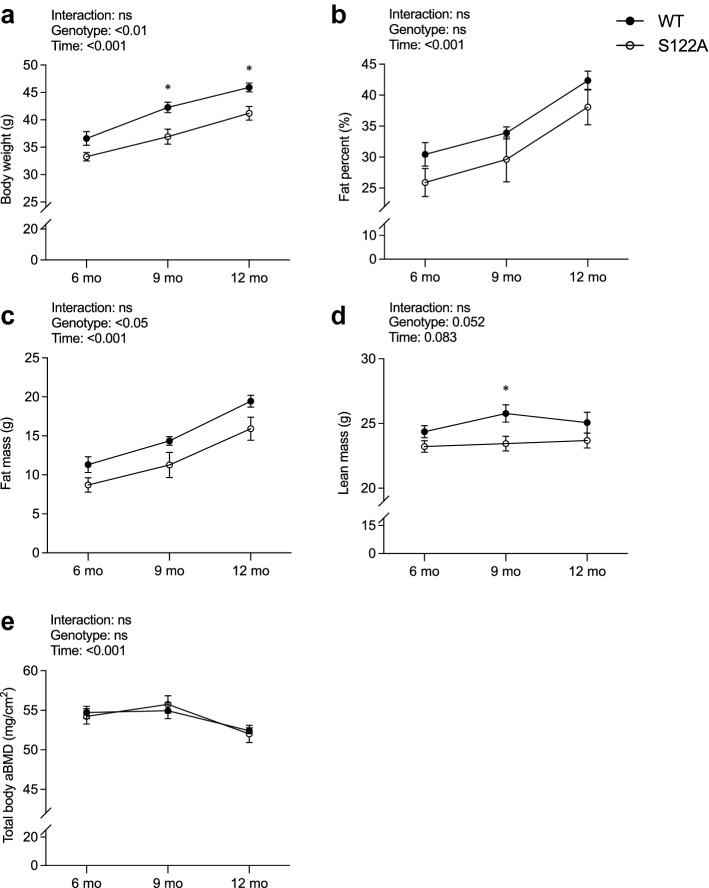
S122A male mice have decreased body weight. Longitudinal measurements of body weight (**a**), lean mass (**b**), fat percent (**c**), fat mass (**d**), and total body aBMD (**e**) by DXA in gonadal intact S122A male mice (n = 9) and WT littermates (n = 10) at six, nine, and twelve months of age. Two-way ANOVA followed by Šidák’s multiple comparisons test.^*^p < 0.05 vs WT at respective timepoint. Data are presented as mean ± sem.

To determine the importance of phosphorylation site S122 in middle-aged males, the mice were analyzed longitudinally with DXA at six, nine, and twelve months of age. Body weight measurements showed that middle-aged (six to twelve months old) S122A male mice have decreased body weight compared to WT littermates (p < 0.01, two-way ANOVA, Fig. 1a), and posthoc analysis revealed significantly decreased body weights in S122A males at nine (− 12%, p < 0.05), and twelve (− 10%, p < 0.05) months of age compared to WT littermates (Fig. 1a). DXA analyses did not reveal any significant differences in fat percent between middle-aged S122A mice and WT littermates (Fig. 1b), while the total body fat mass was significantly decreased in S122A males compared to WT littermates (p < 0.05, two-way ANOVA, Fig. 1c). In addition, there was a tendency to decreased serum leptin levels in S122A males compared to WT littermates at twelve months of age (− 25%, p = 0.055, Table 2). There was a tendency to decreased lean mass in middle-aged S122A males compared to WT littermates (p = 0.052, two-way ANOVA, Fig. 1d), and posthoc analysis demonstrated a transient decrease in lean mass at 9 months of age (− 9%, p < 0.05). Skeletal analysis by DXA showed no significant difference between middle-aged S122A males and WT littermates (Fig. 1e). Consistent with the skeletal DXA results, cortical thickness and trabecular BMD in tibia, measured by pQCT, at the age of 12 months was similar between S122A male mice and WT littermates (Table 2). Analysis of the weights of liver, thymus, testes, and gonadal fat per body weight at twelve months of age did not show any significant differences between S122A male mice and WT littermates (Table 2).

**Table 2:** Description of twelve-month-old male S122A (n = 9) and wild-type (WT, n = 10) littermates.

	WT	S122A
Leptin (ng/ml)	52.1 ± 3.5	39.3 ± 4.9^p=0.055^
Tibia length (mm)	18.8 ± 0.1	18.9 ± 0.1
Tibia cortical thickness (μm)	205.6 ± 6.5	211.0 ± 2.4
Tibia trabecular BMD (mg/cm^3^)	188.0 ± 8.3	206.9 ± 19.4
Liver weight/bw (mg/g)	51.8 ± 3.0	45.3 ± 1.6
Thymus weight/bw (mg/g)	0.90 ± 0.04	0.77 ± 0.04
Testis weight/bw (mg/g)	5.1 ± 0.2	5.3 ± 0.2
Gonadal fat weight/bw (mg/g)	52.1 ± 2.7	58.6 ± 2.6

Serum leptin levels were measured by ELISA. Bone parameters (trabecular BMD and cortical thickness) were analysed by pQCT. Values are given as mean ± SEM. Student's t-test, WT vs S122A mice. bw; body weight.

### S122A male mice have a reduced response to estrogen treatment in cortical bone

**Figure 2:**
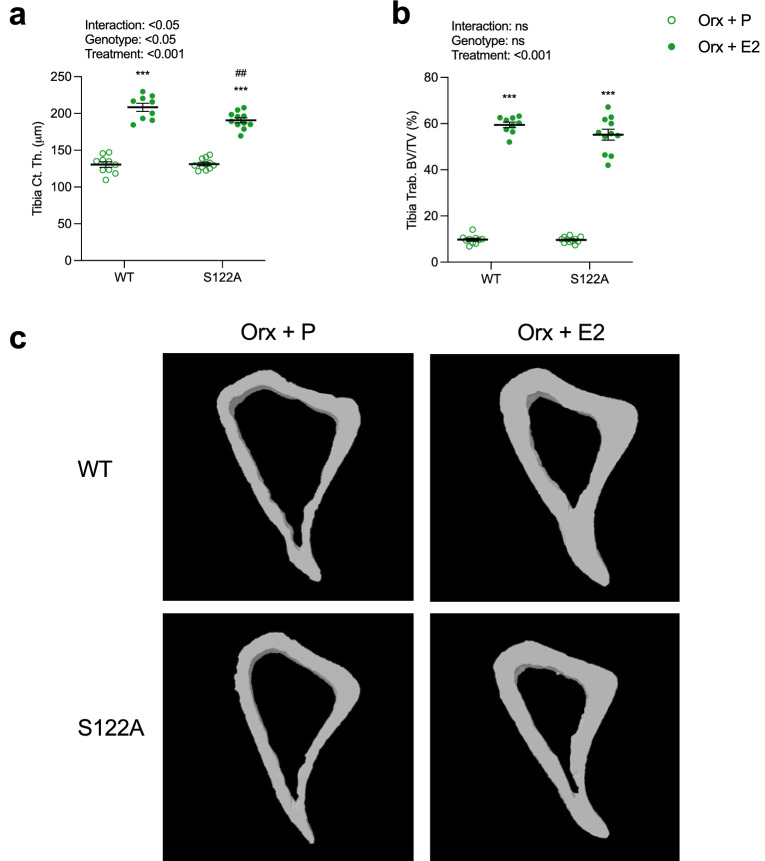
Decreased estrogenic response in cortical bone of S122A male mice. Three-month-old S122A and WT male mice were orx and treated with E2 pellet (16.7 ng/mouse·day) (WT, n = 9; S122A n = 11) or corresponding placebo (P) (WT, n = 10; S122A n = 11) pellet for four weeks. Cortical thickness (Ct.Th) (**a**) and trabecular bone volume per tissue volume (Trab. BV/TV) (**b**) of the tibia were analyzed by high resolution µCT. Representative images of cortical bone from high resolution µCT (**c**). Two-way ANOVA followed by Šidák´s multiple comparisons test. ***p < 0.001 vs Orx + P in respective genotype,^##^p < 0.01 Orx + E2 in S122A versus Orx + E2 in WT. All individual values are presented with mean (horizontal line) and SEM (vertical lines).

To investigate the role of phosphorylation site S122 for the E2 treatment response in bone in males, orx S122A and WT mice were treated subcutaneously with slow-release E2 or placebo pellets for four weeks. As expected, E2 treatment significantly increased the total body aBMD in orx WT compared placebo treatment (+ 25%, p < 0.001, Table 3). E2 treatment also resulted in an increase in total body aBMD in S122A mice (+ 20%, p < 0.001, Table 3), however, the aBMD in E2 treated S122A mice was significantly decreased compared to aBMD in E2 treated WT mice (− 5.1%, p < 0.01, two-way ANOVA followed by Šidák’s multiple comparison test, Table 3). Furthermore, there was a trend towards decreased E2 response in orx S122A males compared to orx WT mice (− 20%, p = 0.06, interaction factor from two-way ANOVA, Table 3). A detailed analysis of trabecular and cortical bone in tibia was performed using µCT to further characterize the responses to E2 treatment in the skeleton of orx S122A male mice. E2 treatment increased cortical thickness in both orx S122A and WT mice, but the cortical thickness was 8.5% lower in the E2 treated S122A compared to WT mice (Fig. 2a, c). Furthermore, the estrogenic response in orx S122A males was significantly decreased compared to the estrogenic response in orx WT males (− 24%, p < 0.05, interaction factor from two-way ANOVA, Fig. 2a, c). The decrease in endosteal circumference after E2 treatment was not significantly different between orx WT and S122A male mice, and no significant E2 treatment effects on periosteal circumference were detected (Table 3). Detailed analysis of trabecular bone parameters using µCT showed that E2 treatment increased trabecular BV/TV and trabecular number, and decreased trabecular separation, to a similar extent in orx WT and S122A male mice (Fig. 2b, Table 3).

**Table 3:** Bone parameters in four-month-old S122A and wild-type (WT) littermates orchidectomized (orx) at 3 months of age and treated with estradiol (E2) (WT, n = 9; S122A, n = 11) or placebo (P) (WT, n = 10; S122A, n = 11) by subcutaneous pellets for 4 weeks.

	WT		S122A		Treatment	Genotype	Interaction
	Orx + P	Orx + E2	Orx + P	Orx + E2			
**DXA**							
Total body aBMD (mg/cm^2^)	48.5 ± 0.3	60.7 ± 1.3***	48.0 ± 0.5	57.7 ± 0.5***^##^	p < 0.001	p < 0.05	0.063
**μCT**							
Endosteal circumference (mm)	4.9 ± 0.1	4.2 ±0.1***	4.8 ± 0.1	4.3 ± 0.1**	p < 0.001	ns	ns
Periosteal circumference (mm)	5.7 ± 0.1	5.5 ± 0.1	5.6 ± 0.1	5.5 ± 0.1	ns	ns	ns
Trabecular thickness (μm)	43.8 ± 1.1	44.1 ± 0.8	43.4 ± 0.8	44.6 ± 0.9	ns	ns	ns
Trabecular number (1/mm)	2.2 ± 0.1	13.5 ± 0.3***	2.2 ± 0.1	12.5 ± 0.7***	p < 0.001	ns	ns
Trabecular separation (μm)	132.2 ± 0.7	46.6 ± 2.1***	132.8 ± 0.3	54.1 ± 5.0***	p < 0.001	ns	ns
**Three-point bending**							
Stiffness (N/mm)	119.9 ± 7.1	175.5 ± 12.0***	116.2 ± 7.4	140.5 ± 8.6^#^	p < 0.001	p < 0.05	ns
Fmax (N)	15.7 ± 0.8	25.4 ± 2.0***	17.0 ± 0.7	22.5 ± 1.3**	p < 0.001	ns	ns

Values are given as mean ± SEM. [n = 9–11]. Two-way ANOVA followed by Šidák´s multiple comparisons test. **p < 0.01, ***p < 0.001 versus Orx + P in WT and S122A mice respectively.^#^p < 0.05,^##^p < 0.01 Orx + E2 in S122A versus Orx + E2 in WT. ns; not significant, Fmax; max load at failure.

To further characterize the role of phosphorylation site S122 of ERα for the response to E2 treatment on the mechanical properties of cortical bone, a three-point bending test was performed on tibia. Compared with placebo, E2 treatment resulted in increased max load at failure and stiffness in WT mice (+ 62% and + 46%, respectively, p < 0.001, Table 3). These parameters were also significantly increased after E2 treatment in S122A males (max load at failure; + 32%, p < 0.01, stiffness; + 21%, p < 0.05, Table 3), but there were tendencies to decreased E2 responses for both max load at failure and stiffness (− 48%, p = 0.1, − 55%, p = 0.08, respectively, interaction factor from two-way ANOVA) when comparing S122A mice to WT littermates (Table 3). Furthermore, the stiffness in E2 treated S122A mice was significantly decreased compared to the stiffness in E2 treated WT mice (− 20%, p < 0.05, two-way ANOVA followed by Šidák’s multiple comparison test, Table 3). In addition, the stiffness of the tibia was significantly decreased in S122A males compared to WT littermates (p < 0.05, two-way ANOVA, genotype effect, Table 3).

A sham group was included in the treatment experiment to determine if the skeletal effects of orx differed between WT and S122A males. Analyses using DXA and µCT showed no differences between sham-operated WT and S122A males (Supplemental Table 1), and orx effects on total body aBMD and cortical and trabecular bone parameters were similar between WT and S122A males (Supplemental Table 2).

### S122A male mice have a normal response to estrogen treatment in non-skeletal tissues

To determine if the E2 response in non-skeletal tissues is affected by the lack of phosphorylation at site S122 in ERα, a DXA analysis was performed to assess effects on lean mass and fat, and several estrogen-responsive tissues were weighed. Comparison of E2 effects on lean mass and fat revealed no differences in treatment response between WT and S122A littermates, and the E2 response on weights per body weights for liver, thymus and gonadal fat were also similar between WT and S122A males (Table 4). In addition, the effect of orx on all evaluated non-skeletal parameters were similar between WT and S122A males (Supplemental Table 2).

**Table 4:** Organ weights and body composition in four-month-old S122A and wild-type (WT) littermates orchidectomized (orx) at 3 months of age and treated with estradiol (E2) (WT, n = 9; S122A, n = 11) or placebo (P) (WT, n = 10; S122A, n = 11) by subcutaneous pellets for 4 weeks.

	WT		S122A		Treatment	Genotype	Interaction
	Orx + P	Orx + E2	Orx + P	Orx + E2			
Body weight (g)	25.8 ± 0.5	27.1 ± 0.8	25.3 ± 0.5	26.2 ± 0.6	ns	ns	ns
Liver weight/bw (mg/g)	44.2 ± 1.0	47.6 ± 0.8*	43.6 ± 1.0	48.1 ± 0.9**	p < 0.001	ns	ns
Thymus weight/bw (mg/g)	3.4 ± 0.1	1.3 ± 0.1***	3.4 ± 0.1	1.6 ± 0.2***	p < 0.001	ns	ns
Gonadal fat weight/bw (mg/g)	16.1 ± 1.4	10.5 ± 1.4**	18.1 ± 1.2	11.7 ± 0.9***	p < 0.001	ns	ns
Lean mass (g)	20.5 ± 0.5	22.1 ± 0.8	20.0 ± 0.4	21.0 ± 0.5	p < 0.05	ns	ns
Fat percent (%)	15.5 ± 0.8	12.9 ± 0.6**	15.8 ± 0.4	13.5 ± 0.5*	p < 0.001	ns	ns
Fat mass (g)	4.0 ± 0.2	3.5 ± 0.2	4.0 ± 0.2	3.5 ± 0.1	p < 0.01	ns	ns

Values are given as mean ± SEM. [n = 9–11]. Two-way ANOVA followed by Šidák’s multiple comparisons test, *p < 0.05, **p < 0.01, ***p < 0.001 versus Orx + P in WT and S122A mice respectively. bw; body weight, ns; not significant.

## Discussion

Estrogen has long been considered to be a female sex hormone important for female reproduction, but it also has major effects in several non-reproductive tissues not only in females but also in males. One of these tissues is the skeleton. A protective role of estrogen for the skeleton in females is well established, and the importance for the male skeleton is being increasingly recognized. However, to use estrogen as a treatment against bone loss in males is not an option due to feminizing effects and other adverse effects in non-skeletal tissues. To benefit from the protective effects of estrogen in males and develop tissue-selective treatment strategies, more knowledge regarding the mechanism behind the effects of estrogen on the male skeleton and other tissues is needed. In this study we show that mutation of phosphorylation site S122 in ERα decreases the response to estrogen treatment specifically in cortical bone compared to WT littermates, while the estrogen responses in other estrogen-responsive tissues are unchanged between S122A and WT mice. Thus, targeting phosphorylation site S122 in ERα can result in tissue-selective, bone-specific, effects in males.

Analysis of young adult gonadal intact S122A males did not reveal any apparent phenotype. Three-month-old S122A males displayed normal body weight, lean mass and total body fat mass as compared to WT littermates. In addition, all body-weight related tissue weights examined were also similar in S122A males compared to WT littermates. Since ERα signaling is involved in the sex steroid feedback regulation in both males and females^22,38,39^, we measured serum levels of sex hormones. There were no significant differences in serum sex steroid levels between S122A and WT male mice, similarly as seen in females^34^, suggesting that the sex steroid feedback is normal in both female and male mice lacking phosphorylation site S122 in ERα. Interestingly, when analysing older, middle-aged males, there was a significant decrease in body weight in S122A compared to WT mice. The bone lengths were similar between S122A and WT males at 12 months of age, indicating that the decrease in body weight is not caused by a general growth retardation. The decreased body weight was accompanied by a significant decrease in total body fat mass in middle-aged males. However, the weight of dissected gonadal fat per body weight was not affected by the S122A mutation, indicating that other fat depots, such as subcutaneous fat or mesenteric fat, may be altered in S122A males compared to WT mice. Further studies are needed to clarify the relationship between phosphorylation of ERα site S122 and fat mass in middle-aged male mice.

In addition to our model investigating the role of a phosphorylation-site in ERα in vivo, two other mouse models have been described^40,41^. Manipulation of ERα signaling is known to affect the body weight of both males and females^38,42^, and in one of the phosphorylation-site models, where a change in serine 216 to alanine results in loss of phosphorylation, body weight is increased in both male and female S216A mice compared to control mice^40^. In the other model, where a Y541S mutation in ERα leads to constitutive activation of the receptor, both male and female mice are growth-retarded and have decreased body weights compared to their controls^41^. Thus, manipulation of phosphorylation sites 216 and 541 in the murine ERα results in similar effects on body weight in females and males. Interestingly, in our model, the lower body weight observed in S122A males compared to littermate controls is opposite to what we reported for the female S122A mice, which had increased body weight compared to littermate controls^34^. These results suggest that phosphorylation of site S122 in ERα might be involved in establishment of gender difference in body weight in mice.

Analyses of the skeleton of gonadal intact young-adults and middle-aged male mice revealed no statistically significant differences between S122A and WT mice. Thus, phosphorylation of site S122 in ERα does not seem to be required for regulation of the skeleton in males under normal physiological conditions. Total body aBMD in E2 treated S122A mice was significantly decreased compared to E2 treated WT mice, and the pharmacological E2 treatment resulted in a trend of decreased E2 response in total aBMD in S122A compared with WT as measured by DXA, which does not separate the trabecular and cortical bone compartments. More detailed analysis of the tibia with μCT showed that there are no differences in the E2 response on trabecular bone parameters between S122A males and WT littermates. However, examination of the cortical bone showed that the increase in thickness after E2 treatment was significantly lower in magnitude in S122A mice compared to WT males. Thus, the ability to phosphorylate site S122 in ERα is required for a normal response to E2 treatment in the cortical bone compartment. Approximately 80% of the total skeletal bone mass is cortical bone, and this bone compartment is important for skeletal strength^43^. Although there were no statistically significant differences in the E2 responses between S122A and WT males when analysing mechanical properties using three-point bending, we found a clear tendency to decreased E2-response for stiffness. In addition, the stiffness in the E2 treated S122A mice was significantly decreased compared to the stiffness in E2 treated WT mice. Together these data suggest that phosphorylation of S122 in ERα might be an important target for increasing cortical bone mass and bone strength in males. Importantly, the mutation of phosphorylation site S122 in ERα did not alter ERα mRNA expression in cortical bone in males, similar to previous observations in females^34^.

Estrogen treatment not only affects the skeleton in male mice, but also several other tissues. E2 treatment is known to decrease thymus weight^44^ and fat mass in males^45^. Interestingly, the responses to pharmacological E2 treatment were similar in these non-skeletal tissues, as well as in liver, between S122A and WT males. Thus, the importance of phosphorylation site S122 in ERα for the E2 treatment response seems to be specific for the cortical bone compartment.

We have previously shown that the S122A mutation leads to loss of the ability to phosphorylate ERα at site 122^34^. The S122A mutation involves an amino acid change to alanine. Alanine is commonly used as the amino acid substitute in many transgenic animals when a single amino acid change is introduced to learn about the function of a specific site because of its ability to mimic the secondary structure preferences of other amino acids and thereby have a minimal impact on the protein structure^46^. However, the possibility that the amino acid change still might slightly contribute to the phenotype cannot be completely ruled out.

In summary, we show that mutation of phosphorylation site S122 in ERα results in decreased body weight in middle-aged gonadal intact male mice compared to WT littermates. We also show that lacking the phosphorylation site S122 does not affect the skeleton in neither young adult, nor middle-aged gonadal intact male mice. Importantly, we find a decreased response to pharmacologic E2 treatment in cortical bone in orx S122A male mice compared to WT male mice, while the E2 responses are normal between WT and S122A mice in all other tissues examined. This demonstrates that manipulation of the phosphorylation site S122 in ERα affects the response to E2 treatment in a tissue-dependent manner in males, specifically targeting cortical bone. This knowledge may aid the development of novel estrogenic drugs against bone loss in men.

## Supplementary Information

Supplementary Tables.

## Data Availability

The data that support the findings of this study are available in the methods, results, and supplementary material of this article and in figshare repository, 10.6084/m9.figshare.21564717.
