# Supplementary material for: Phosphorylation of S122 in ERα is important for the skeletal response to estrogen treatment in male mice
Source: Sci Rep. 2022 Dec 27;12:22449. doi: 10.1038/s41598-022-26939-9 (PMC9794719; doi:10.1038/s41598-022-26939-9)
Supplement: Supplementary file 1 — Supplementary Tables. [file 41598_2022_26939_MOESM1_ESM.pdf]

Supplemental Table 1

	WT	S122A
DXA		
Total body aBMD (mg/cm^2)	53.3 ± 0.8	53.5 ± 0.6
uCT Tibia		
Cortical thickness (µm)	157.3 ± 3.5	159.0 ± 3.3
Endosteal circumference (mm)	4.7 ± 0.1	4.7 ± 0.1
Periosteal circumference (mm)	5.7 ± 0.1	5.7 ± 0.1
Trabecular BV/TV (%)	21.4 ± 1.5	22.8 ± 1.3
Trabecular thickness (µm)	46.6 ± 1.3	47.8 ± 1.2
Trabecular number (1/mm)	4.6 ± 0.3	4.7 ± 0.2
Trabecular separation (µm)	118.4 ± 2.1	116.6 ± 1.4
Three point bending		
Stiffness (N/mm)	134.6 ± 14.5	142.4 ± 9.5
Fmax (N)	21.5 ± 2.0	22.0 ± 1.3
Body weight (g)	28.6 ± 0.6	28.2 ± 0.6
Liver weight/bw (mg/g)	48.7 ± 1.7	48.3 ± 1.0
Thymus weight/bw (mg/g)	1.3 ± 0.1	1.1 ± 0.1
Gonadal fat weight/bw (mg/g)	21.2 ± 2.7	19.0 ± 2.7
Lean mass (g)	22.6 ± 0.6	22.1 ± 0.5
Fat percent (%)	18.0 ± 1.7	17.5 ± 1.4
Fat mass (g)	5.2 ± 0.5	5.0 ± 0.5

Description of four-month-old S122A (n=11) and wild-type (WT) littermates (n=8) sham-operated at three months of age and treated with placebo pellets for four weeks. Values are given as mean ± SEM. Student's t-test, WT vs S122A mice. bw; body weight.

Supplemental Table 2

	WT (%)	S122A (%)	Sham/Orx	Genotype	Interaction
DXA					
Total body aBMD (mg/cm^2)	-9.0 ± 0.6***	-10.3 ± 0.8***	p<0.001	ns	ns
uCT Tibia					
Cortical thickness (µm)	-17.1 ± 2.4***	-17.5 ± 1.4***	p<0.001	ns	ns
Endosteal circumference (mm)	3.3 ± 2.3	1.1 ± 1.7	ns	ns	ns
Periosteal circumference (mm)	-0.3 ± 1.6	-2.2 ± 1.3	ns	ns	ns
Trabecular BV/TV (%)	-54.5 ± 2.9***	-57.7 ± 1.7***	p<0.001	ns	ns
Trabecular thickness (µm)	-6.2 ± 2.3	-9.3 ± 1.7*	p<0.01	ns	ns
Trabecular number (1/mm)	-51.6 ± 2.3***	-53.2 ± 1.4***	p<0.001	ns	ns
Trabecular separation (µm)	11.6 ± 0.6***	13.9 ± 0.3***	p<0.001	ns	ns
Three point bending					
Stiffness (N/mm)	-10.9 ± 5.2	-18.4 ± 5.2	p<0.05	ns	ns
Fmax (N)	-26.9 ± 3.7**	-22.5 ± 3.3*	p<0.001	ns	ns
Body weight (g)	-10.0 ± 1.6**	-10.6 ± 1.8***	p<0.001	ns	ns
Liver weight/bw (mg/g)	-9.2 ± 2.1*	-9.8 ± 2.0**	p<0.001	ns	ns
Thymus weight/bw (mg/g)	172 ± 10***	192 ± 10***	p<0.001	ns	ns
Gonadal fat weight/bw (mg/g)	-23.9 ± 6.8	-4.5 ± 6.5	ns	ns	ns
Lean mass (g)	-9.2 ± 2.1**	-9.4 ± 1.7**	p<0.001	ns	ns
Fat percent (%)	-13.9 ± 4.4	-9.8 ± 2.4	ns	ns	ns
Fat mass (g)	-23.1 ± 3.5	-19.8 ± 3.1	p<0.01	ns	ns

Effects of orchidectomy (orx) on bone parameters, organ weights and body composition presented as percent change between orx (WT, n=10 ; S122A, n=11) and sham-operated (WT, n=8; S122A, n=11) mice. Values are given as mean±SEM. Two-way ANOVA followed by Šidák´s multiple comparisons test, $^{*}p<0.05$, $^{**}p<0.01$, $^{***}p<0.001$ versus Orx + P in WT and S122A mice respectively. Orx; orchidectomy, ns; not significant, bw; body weight.
